# Interleukins in Platelet Biology: Unraveling the Complex Regulatory Network

**DOI:** 10.3390/ph17010109

**Published:** 2024-01-13

**Authors:** Miao Huang, Long Wang, Qianhui Zhang, Ling Zhou, Rui Liao, Anguo Wu, Xinle Wang, Jiesi Luo, Feihong Huang, Wenjun Zou, Jianming Wu

**Affiliations:** 1 State Key Laboratory of Southwestern Chinese Medicine Resources, School of Pharmacy, Chengdu University of Traditional Chinese Medicine, Chengdu 611137, China; hm80918885@126.com (M.H.); 13098519311@163.com (Q.Z.); 2 Department of Pharmacology, School of Pharmacy, Southwest Medical University, Luzhou 646000, China; wanglongsdu1226@163.com (L.W.); zhouling@stu.swmu.edu.cn (L.Z.); liao1908651016@163.com (R.L.); wag1114@foxmail.com (A.W.); huangfeihong@swmu.edu.cn (F.H.); 3 Department of Physiology, School of Basic Medical Sciences, Southwest Medical University, Luzhou 646000, China; cqwmn@sina.com (X.W.); ljs@swmu.edu.cn (J.L.); 4 The Key Laboratory of Medical Electrophysiology, Institute of Cardiovascular Research, Ministry of Education of China, Luzhou 646000, China

**Keywords:** interleukins, megakaryopoiesis, thrombopoiesis, bioengineering

## Abstract

Interleukins, a diverse family of cytokines produced by various cells, play crucial roles in immune responses, immunoregulation, and a wide range of physiological and pathological processes. In the context of megakaryopoiesis, thrombopoiesis, and platelet function, interleukins have emerged as key regulators, exerting significant influence on the development, maturation, and activity of megakaryocytes (MKs) and platelets. While the therapeutic potential of interleukins in platelet-related diseases has been recognized for decades, their clinical application has been hindered by limitations in basic research and challenges in drug development. Recent advancements in understanding the molecular mechanisms of interleukins and their interactions with MKs and platelets, coupled with breakthroughs in cytokine engineering, have revitalized the field of interleukin-based therapeutics. These breakthroughs have paved the way for the development of more effective and specific interleukin-based therapies for the treatment of platelet disorders. This review provides a comprehensive overview of the effects of interleukins on megakaryopoiesis, thrombopoiesis, and platelet function. It highlights the potential clinical applications of interleukins in regulating megakaryopoiesis and platelet function and discusses the latest bioengineering technologies that could improve the pharmacokinetic properties of interleukins. By synthesizing the current knowledge in this field, this review aims to provide valuable insights for future research into the clinical application of interleukins in platelet-related diseases.

## 1. Introduction

Megakaryocytes (MKs), mature hematopoietic cells derived from hematopoietic stem cells (HSCs), constitute a mere 0.05% of the total nucleated cells in bone marrow. Despite their scarcity, platelets play a pivotal role in a variety of physiological and pathological processes, including hemostasis, inflammation, and immunity [1]. The normal platelet counts in human blood range from 150 to 450 × 10^9^/L. The human body releases approximately 100 billion platelets daily to maintain the standard quantity and functionality of platelets [2]. Any abnormality in this process of platelet homeostasis can severely impact the number or function of platelets, leading to platelet disorders [3]. Platelet disorders can be categorized into two main types based on their origin: acquired and inherited platelet disorders, which manifest as thrombocytosis, thrombocytopenia, and platelet function disorders. Acquired platelet disorders arise from external factors such as infections, medications, or autoimmune diseases, while inherited platelet disorders are caused by genetic mutations [4,5]. The severity of these disorders can vary dramatically in clinical settings. Even within the same type of disorder, the severity can range from negligible to life-threatening [6]. Some platelet disorders, due to their complexity and specificity, remain difficult to treat effectively as a universally effective cure has yet to be discovered [7]. The comprehensive study of the physiology of HSCs, their development into MKs, and subsequent platelet production and regulation is crucial for modern researchers to understand and investigate the corresponding pathological processes of platelet disorders. There are numerous fundamental studies in this area, and the traditional pathway of HSCs gradually developing into MKs in a homeostatic environment is largely understood. In recent years, the process of MK formation under dynamic and adaptive biological demands has been elucidated, revealing a non-classical pathway of MK development directly from HSCs [8]. These pathways are influenced and regulated by various cytokines, with interleukins playing a significant role.

Interleukins, cytokines secreted by various cells such as macrophages, lymphocytes, and MKs, are pivotal participants in immune responses and immunoregulation, among other physiological processes [9,10]. Furthermore, they also play a role in regulating a wide range of physiological and pathological reactions within the body [11]. Modern research has discovered that various interleukins play significant and unique roles in regulating the development and function of MKs and mediate the development of a range of platelet disorders [12]. Utilizing the regulation of interleukins to achieve therapeutic effects on related diseases is a promising clinical strategy. However, only a limited number of interleukin biologics are currently approved for clinical use. Among interleukin receptor agonists, IL-11 is predominantly used to stimulate megakaryopoiesis for the treatment of chemotherapy-induced thrombocytopenia. Indeed, the interleukin family holds many untapped treasures. Summarizing the effects of interleukins on MK lineage cells and understanding their mechanisms of action is essential. We used the terms “interleukin”, “IL”, “megakaryocytes”, and “platelets” via Google Scholar, PubMed, and Web of Science, covering the period 1987–2023. After excluding some literature based on their titles and abstracts, the literature related to the effects and mechanisms of interleukins on MK lineage cells was included by reading the full text of the literature. A deeper comprehension of the significant roles that interleukins play in regulating the development and function of MK lineage cells in both healthy and pathological states could potentially pave the way for discovering new clinical methods for treating platelet-related diseases via interleukin regulation.

## 2. Overview of Hematopoiesis and Megakaryopoiesis

Hematopoiesis, the process of differentiation of HSCs into various lineages, is a vital physiological process in humans. HSCs differentiate into myeloid and lymphoid progenitor cells, which in turn differentiate into various cell types such as erythroid, leukocyte, and MK lineages [13]. These cells are essential for maintaining normal body functions.

The traditional view of the hematopoietic system posits that long-term hematopoietic stem cells (LT-HSCs) reside at the pinnacle of the hematopoietic hierarchy. Upon activation, these cells generate more dynamic short-term hematopoietic stem cells (ST-HSCs). These stem cells undergo a stringent differentiation process and balanced lineage progression, ultimately resulting in the generation of all types of blood cells [14]. Taking the differentiation of the MK lineage as an example, HSCs undergo several stages, including multipotent progenitor (MPP), common myeloid progenitors (CMP), megakaryocyte-erythroid progenitor (MEP), and megakaryocyte progenitor (MKP) [15]. With each stage, the potential for lineage differentiation is progressively lost. This process leads to the formation of immature MKs, which then undergo a series of cytoplasmic and nuclear maturation processes. Ultimately, these immature MKs develop into mature MKs with the capability to release platelets [16,17]. However, this theoretical framework has recently been called into question. Some researchers contend that the classical model of a hematopoietic hierarchy, based on in vitro transplantation, does not fully encapsulate the actual process of HSC lineage differentiation in vivo. With the advent of single-cell cytogenomics and novel lineage tracing technologies in hematopoietic research, there is mounting evidence that the differentiation process of hematopoietic stem and progenitor cells (HSPCs) in vivo does not adhere strictly to the classical model [18]. Consequently, an updated comprehension of this intricate process is warranted.

In recent years, discoveries have unveiled the heterogeneity of HSCs. This is evidenced by the observation that different subpopulations of HSCs exhibit a bias toward lineage differentiation. This suggests that the outcome of certain cell lineage differentiation may be predetermined as early as the stem cell stage. Furthermore, it implies that different subpopulations of stem cells, each with different differentiation potentials, may be responsible for major hematopoiesis in varying physiological environments [19,20,21]. Some studies have proposed that the regulation of HSC lineage differentiation may be influenced by the ecological niche in which the HSCs reside. Different ecological niches categorize HSCs into distinct subpopulations, which in turn determine the stimuli that the stem cells can receive. This, to some extent, also influences the bias of HSC lineage differentiation. In other words, HSCs situated in a specific ecological niche are only capable of receiving particular stimuli and then differentiate toward the lineage to which they are biased [22,23]. Moreover, a recent study revealed the origin of heterogeneity in zebrafish embryonic hematopoietic stem progenitor cells. Using single-cell transcriptome analysis, lineage tracing, and functional assays, the researchers demonstrated that the heterogeneity of HSPCs is derived from heterogeneous hemogenic endothelial cells (HECs) and found that the transcription factor Spi2 is required for the formation of lymphoid/myeloid-primed HSPCs by tightly controlling the endothelial-to-hematopoietic transition program. Their study suggests that lineage competence is conferred earlier in embryonic development than previously thought and identifies a new molecular determinant of lineage-biased HSPC generation in vertebrates [24].

Among the numerous studies on HSC lineage bias, it has been found that the MK lineage is unique in its ability to develop directly from LT-HSCs. This process bypasses the pluripotent progenitor cell stage, enabling rapid differentiation into MKs [25]. The MK-biased HSCs from which they originate represent the only subpopulation of stem cells that exhibit a monolineage bias. This differentiation process serves as the primary pathway for the emergency generation of MKs in response to inflammation or severe thrombocytopenia, which may be related to the fact that MKs have the function of directly regulating HSCs and their ecological niche [26]. Furthermore, the platelet release pattern of emergency MK generation differs from the process of platelet release from proplatelet formation (PPF) under homeostatic conditions. In emergency situations, MKs rupture themselves to rapidly produce large quantities of platelets, thereby meeting the body’s acute demand for platelets [27]. These homeostatic or non-homeostatic thrombocytopoietic processes are co-regulated by a variety of cytokines, and interleukins, in particular, play an important role in megakaryopoiesis.

## 3. Overview of Interleukins

Interleukins, initially identified as products of leukocytes and mediators within this cell population, are now recognized as a subset of cytokines. These molecules are synthesized by a broad array of cells, and their molecular structure and biological functions have been largely elucidated. They play pivotal regulatory roles in cellular interactions and immune responses [28,29]. The study of interleukins has a history dating back to the 1940s. However, it was not until 1979 that the first two interleukins were identified and officially named. These were the Lymphocyte Activating Factor (LAF) and the T-cell Growth Factor (TCGF), which are now known as IL-1 and IL-2, respectively [30]. Since then, the guidelines for naming interleukins have been refined, and to date, no fewer than 40 interleukins have been identified and named [31]. The discovery of new members continues at a consistent pace. Contemporary researchers have classified interleukins into families based on their receptor chain similarity, sequence homology, and functional characteristics. Examples include the interleukin-1 family (IL-1α, IL-1β, IL-18, IL-33, IL-36α, IL-36β, IL-36γ, IL-37, and IL-38); interleukin-2 (common γ-chain) family (IL-2, IL-4, IL-7, IL-9, IL-15, and IL-21); interleukin-17 family (IL-17A/F, IL-17B, IL-17C, IL-17D, and IL-25); and others [29,32,33,34].

Interleukins, like other cytokines, have a wide range of functions and properties; they exert their influence by binding to receptors located on the cell membrane. This binding triggers specific pathways within the cell that promote the synthesis of target proteins, a process that ultimately facilitates the profound regulation of cellular function [35]. The primary role of interleukins is to mediate the immune response and regulate the growth, differentiation, and activation of other immune cells, and they can trigger a variety of responses, including inflammation, proliferation, maturation, migration, and adhesion. The relationship between interleukins and MKs has long captured researchers’ attention. Starting in the 1980s, studies investigating how interleukins regulate MK differentiation and function emerged. From 1990 to 2000, research activity in this area surged, with a specific emphasis on the main effects and mechanisms of three cytokines: IL-3, IL-6, and IL-11 [36,37,38]. These cytokines play a critical role in megakaryopoiesis and have led to significant discoveries during that period. In recent years, developments in the process of HSC differentiation into MKs, the discovery and understanding of new interleukins, and fresh insights into existing ones have revitalized research in this area. Moreover, significant progress in cytokine engineering has intensified the focus on the regulatory function of interleukins in MK differentiation and function. At the same time, as our understanding of MKs grows, their crucial role in the immune system has gained recognition. MK and platelets are now widely acknowledged as integral parts of the immune system [39,40]. Consequently, the impact of interleukins on MKs, which act as major regulators in immune cell development, has received elevated attention.

## 4. The Role of Interleukins in Thrombopoiesis

**Figure 1:**
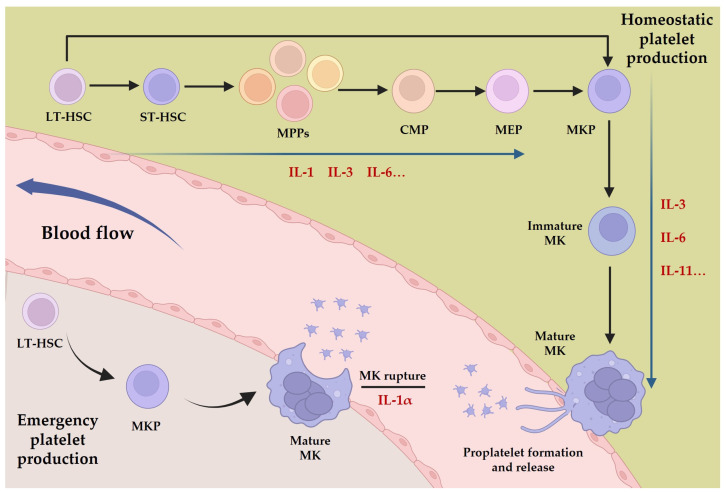
Platelet production and the interleukins involved in the process. Depending on the state of the body, platelets have two generative processes, homeostatic platelet production, and emergency platelet production, and interleukins are involved in different stages of these processes, such as IL-1, IL-3, IL-6, IL-11, etc.

The intricate process of platelet production, known as thrombopoiesis, is tightly regulated and involves the differentiation and maturation of MKs. It can be divided into three distinct stages. Initially, LT-HSCs are activated from a quiescent state to generate more active ST-HSCs; these then undergo a gradual loss of pluripotency and differentiate into MKPs via the stages of MPPs, CMPs, and MEPs. Subsequently, the progenitor cells proliferate and differentiate into immature MKs, which then undergo a series of maturation processes, such as an increase in DNA content, acquisition of surface proteins, and formation of the demarcation membrane system to generate mature MKs [41]. Finally, mature MKs either release platelets via a process called proplatelet formation or undergo rupture to release platelets directly into the bloodstream. Multiple cytokines, including thrombopoietin (TPO), play crucial roles in regulating thrombopoiesis at all stages, with TPO serving as the primary cytokine for regulating the process mentioned above [42]. Other cytokines, such as stem cell factor (SCF), granulocyte-macrophage colony-stimulating factor (GM-SCF), stromal cell-derived factor 1 (SDF-1), and chemokine ligand 5 (CCL5), also contribute to the process of MK development and platelet production [43,44,45,46]. Interleukins, a family of signaling molecules, have emerged as critical regulators of thrombopoiesis. Interleukins such as IL-1, IL-3, and IL-6 promote the differentiation of HSCs into MKPs in the early stage of megakaryopoiesis, while IL-3, IL-6, and IL-11 facilitate the transition from immature to mature MKs [47,48]. Additionally, IL-1α can mediate MK rupture to accelerate platelet release in situations of a sudden platelet count decrease [49] (Figure 1). The intricacies of interleukin mechanisms in thrombopoiesis encompass interactions with other cytokines and signaling pathways. Specifically, interleukins IL-3, IL-6, and IL-11 require the presence of TPO and its receptor to effectively differentiate pro-megakaryocytes. These interleukins typically exhibit less efficacy individually compared to their synergistic performance with other cytokines, and their mechanisms of action are also associated with the three major pathways involved in TPO-mediated MK differentiation: JAK/STAT, PI3K/AKT, and MAPK/ERK [50,51,52].

While there is no evidence to suggest that interleukins are essential in homeostatic megakaryopoiesis, their significant regulatory role in megakaryopoiesis and the correlation between alterations in interleukin expression and changes in platelet count and function in non-homeostatic environments underscore their importance. In particular, IL-1α mediates the release of platelets via MK rupture during emergency platelet production, highlighting the unique influence of interleukins on the dynamics of megakaryopoiesis. Therefore, a thorough comprehension of the effects and mechanisms of interleukins on MK lineage cells is vital for comprehending cytokine regulation of thrombopoiesis in diverse organismal surroundings. This knowledge is expected to promote the progress of research and clinical application of cytokine therapies and biological agents.

## 5. Effects of Interleukins on Megakaryopoiesis and Platelet Function

In addition to the well-studied interleukins known to play a clear role in MK differentiation, several other interleukins have been identified as regulators of platelet production and function in vitro and in vivo. These interleukins have been classified within the interleukin family, and the sources of these regulatory interleukins in MKs, and their sources, along with their impact on platelet generation and function, are summarized in Table 1.

**Figure 2:**
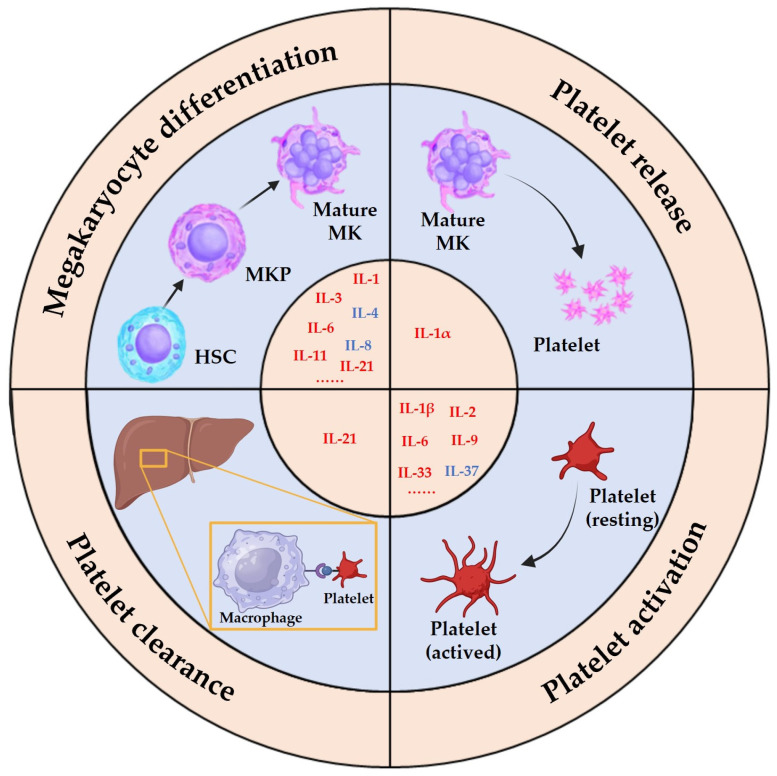
Effects of interleukins on megakaryocyte lineage cells. Interleukins have a variety of effects on megakaryocyte lineage cells in vivo and in vitro, which can be categorized into four main aspects: effects on megakaryocyte differentiation, platelet release, platelet clearance, and platelet function.

The impact of interleukins on the MK lineage cells can be broadly categorized into four aspects: their impact on MK differentiation, their role in platelet release, their effect on platelet function, and their involvement in platelet clearance (Figure 2). While most interleukins exert a promotive effect on MK differentiation and platelet function, a few have inhibitory effects. For instance, IL-1α enhances platelet release, and IL-4 inhibits MK differentiation. Notably, IL-2 has been observed to have variable effects on platelet counts under different pathological conditions. This suggests that interleukins can have diverse effects in different organismal environments. Therefore, comprehensively analyzing the underlying mechanisms of action is essential for understanding the pleiotropic effects of interleukins.

## 6. Mechanisms of Interleukin Effects on Megakaryocyte Lineage Cells

Most cytokines, including interleukins, exert their effects by binding to cell surface receptors. Interleukins typically engage high-affinity receptor subunits on the cell surface, followed by recruiting low-affinity receptor subunits to form receptor complexes. These receptor complexes, in turn, activate multiple signaling pathways in the target cell. In addition to the primary JAK-STAT pathway, certain interleukins also instigate the activation of several other pathways, such as MAPK, PI3K/AKT, and NF-κB [74]. These pathways are also crucial in influencing the development and functional regulation of MKs [75,76,77]. The mechanisms of interleukins in cells of the MK lineage are now summarized by classifying them in terms of interleukin families.

### 6.1. The IL-1 Family and Its Role in Megakaryopoiesis and Platelet Function

Within the IL-1 family, IL-1 stands out as a significant pro-inflammatory cytokine, instigating the immune system to mount an inflammatory response. Its members, IL-1α and IL-1β, exert a multitude of influences on MKs and platelets. Interleukin-1β (IL-1β) enhances the functionality of MKs and platelets in both humans and mice and stimulates the bone marrow to increase megakaryopoiesis. This process activates multiple signaling pathways via IL-1R1, including NF-κB, MAPK/ERK, and PI3K/AKT. As a result, there is an increase in the expression of crucial genes that are important for platelet production and functional activation [54]. Moreover, platelets possess the capability to produce and release IL-1β, establishing an IL-1β autocrine stimulation loop to perpetually manifest its influence [53]. In another study, myocardial infarction induced the upregulation of bone marrow NOD-like receptor protein 3 (NLRP3) and the subsequent secretion of IL-1β, which may contribute to aggravation of atherosclerosis by promoting MK development and platelet production [78].

Beyond its function in PPF-type thrombopoiesis under homeostatic conditions, IL-1α also promotes MK rupture-induced thrombopoiesis in scenarios of inflammation or significant platelet depletion. In response to the immediate requirement for platelet synthesis, IL-1α triggers certain thrombopoiesis and proapoptotic genes via IL-1R1 signaling and activates the phosphorylation of proteins such as Caspase-3, AKT, and ERK, among others, resulting in the rupture of MKs to yield a substantial quantity of platelets. Notably, this mechanism of platelet release differs from the conventional PPF-type platelet production and FasL-induced apoptosis in terms of morphological observation [49,79]. Further investigation reveals that IL-1α’s role in suppressing PPF, inducing MK rupture, and swiftly producing a substantial number of platelets may be associated with the dysregulation of tubulin synthesis, and this could potentially be ascribed to the disruption of microtubule assembly incited by IL-1α [80]. Additionally, a recent study found that IL-1 does not directly activate HSCs in the presence of platelet depletion but instead activates bone marrow Lepr+ perivascular niche cells expressing the IL-1 receptor, which in turn leads to the optimal activation of quiescent HSCs, resulting in their direct differentiation into MKs and the restoration of platelet homeostasis [81].

While IL-33 does not directly influence platelets, the recent literature indicates that intestinal damage and inflammation can stimulate systemic coagulation via an IL-33-dependent process. IL-33 indirectly influences the function of platelets throughout the body by promoting the release of 5-HT from the intestine. This process is linked to the augmentation of 5-HT release from intestinal enterochromaffin cells via the IL-33-ST2 pathway, which impacts whole-body platelet function, as evidenced by the swift activation of platelets for hemostasis and the enhanced capacity to recruit neutrophils [55].

IL-37, in contrast to the pro-inflammatory properties demonstrated by other members of the IL-1 family, plays a distinctive role in the widespread inhibition of inflammation and specific immune responses [82]. Research has identified an increase in IL-37 levels in the plasma of patients suffering from myocardial infarction. These elevated levels have been found to directly inhibit platelet activation and thrombosis in vivo, thereby mitigating cardiac injury post-myocardial infarction. Furthermore, IL-37’s receptor, IL-1R8, is a significant modulator of platelet function. It is highly expressed on platelets, and platelets from IL-1R8 knockout mice have been observed to exhibit enhanced adhesion and aggregation capacity. IL-37 could initiate the IL-1R8/PTEN/(Akt and Syk) signaling pathway by binding to IL-1R8 and IL-18Rα on platelets. This interaction results in an inhibitory effect that mitigates atherosclerotic thrombosis and infarct extension [56]. Therefore, IL-37 presents potential therapeutic advantages as a target for antithrombotic drugs.

### 6.2. The IL-2 Family and Its Role in Megakaryopoiesis and Platelet Function

IL-2, as one of the inaugural cytokines sanctioned by the U.S. Food and Drug Administration for immunotherapeutic applications in oncology, plays a pivotal role in the management of neoplastic and autoimmune disorders, its primary mechanism of action involves modulating the differentiation and functionality of CD4+ T lymphocytes [83]. While the direct influence of IL-2 on platelets remains elusive, preliminary investigations have reported adverse outcomes such as thrombocytopenia with increased viability of residual platelets with moderate and high-dose IL-2 treatment in cancer patients [57]. Conversely, the administration of low-dose IL-2 to adult patients with immune thrombocytopenia (ITP) results in a significant augmentation of T regulatory (Treg) cells and platelets. This mechanism is potentially associated with IL-2’s role in regulating and restoring the equilibrium of the Th17/Treg cellular axis [58,84]. In another in vitro study, it was observed that the administration of IL-2 in liquid cultures lacking monocytes had no direct effect on MKs cultured from CD34+ HSCs. In contrast, the administration of IL-2 in peripheral blood monocyte liquid cultures with abundant hematopoietic progenitor cells results in a significant rise in the number of polyploids in immature MKs developed under identical conditions. Further studies reveal that the observation is related to a particular soluble peptide generated by IL-2 when stimulating natural killer (NK) cells. The authors posit that this peptide could encourage mitosis and increase the ploidy of immature human MKs in vitro in the presence of IL-3 and SCF [85]. However, the article does not reveal the specific identity of the peptide.

IL-4, a pleiotropic cytokine, has been found to exert a predominantly negative regulatory effect on MKs. Early research has demonstrated that it significantly impedes the formation of both pure and mixed MK colonies in a dose-dependent manner in vitro; furthermore, it appears to influence the early stages of MKP proliferation [86]. IL-4 has been shown to inhibit the expression of NF-E2 mRNA and protein in CD34-derived MKs, thereby inhibiting MK maturation and platelet production [60]. Moreover, an increase in IL-4 derived from bone marrow endothelial cells and its subsequent inhibition of bone marrow CFU-MK formation and MK maturation have been observed in mice with acute myeloid leukemia. This mechanism may be associated with the downregulation of the NF-E2 expression and the phosphorylation of STAT6 [59]. These findings suggest that the bone marrow endothelial source of IL-4 could potentially serve as a therapeutic target for acute myeloid leukemia.

**Figure 3:**
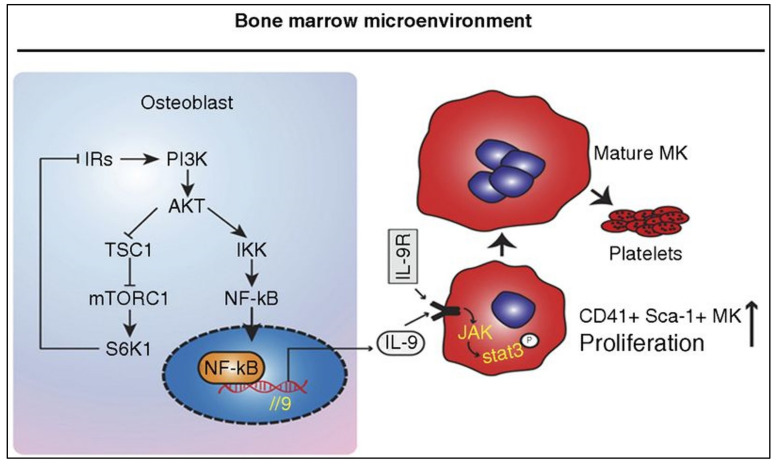
Mechanism of IL-9 produced by osteoblasts facilitates megakaryopoiesis and platelet production. IL-9 can promote CD41+Sca-1+ MK proliferation via the JAK/STAT3 pathway. ↑, Promotation. Reproduced with permission from [62], copyright © 2017 American Society of Hematology.

IL-9 primarily serves as a growth factor for T cells and mast cells. However, its role in the regulation of megakaryopoiesis and maturation has been partially explored. Research has shown that osteoblasts are the primary source of IL-9 in bone marrow. Mouse osteoblasts could activate IL-9R/STAT3 signaling by producing IL-9, promoting CD41+Sca-1+ MK expansion, MK maturation, and thrombopoiesis in vitro and in vivo. The mechanism is also linked to the ability of osteoblasts to regulate the microenvironment of neighboring cells via the production of signaling molecules and paracrine secretion. This regulation can prevent chemotherapy-induced thrombocytopenia and promote platelet recovery in mice at a low dose of IL-9 [62]. In addition, IL-9 could prevent CD41 antibody-induced ITP in BALB/c mice by regulating osteoblasts and activating IL-9R/STAT5 signaling in MKs [87]. In terms of platelet function, it has been shown that platelets express IL-9R and that IL-9 plays an important role in deep vein thrombosis in mice, and it significantly enhances ADP-induced platelet aggregation and P-selectin expression in mice via the JAK2/STAT3 pathway [61] (Figure 3).

**Figure 4:**
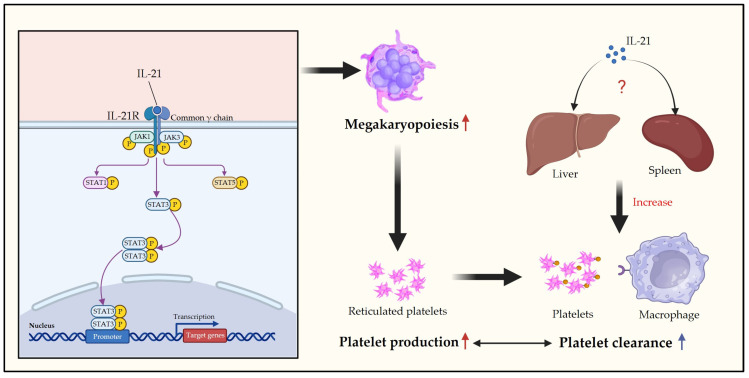
Mechanism of IL-21 regulation of megakaryocyte development and platelet homeostasis. IL-21 promotes megakaryopoiesis via the JAK3/STAT3 pathway to increase platelet production and can maintain platelet homeostasis by increasing platelet clearance in the liver and spleen. ↑, Promotation.

IL-21 acts as an essential immunomodulatory cytokine, regulating the function of various immune cells. Expression of the IL-21R is observed during TPO-induced human MK differentiation, while no expression is detected on human and mouse platelets. This observation suggests that the receptor may only play a role during the MK differentiation stage. Further studies indicate that IL-21 enhances the proliferation of human MKP in vitro in a dose-dependent manner in the presence of TPO, and it promotes MK differentiation and platelet production in mice in vivo via a mechanism that involves JAK3/STAT3 pathway activation. Moreover, IL-21 overexpression in mice stimulates MK development and elevates the number of neonatal platelets without affecting the peripheral platelet levels, potentially due to increased platelet clearance by macrophages in the spleen and liver [63]. These findings demonstrate a bidirectional regulatory effect of IL-21 on platelets, suggesting its involvement in maintaining platelet count homeostasis (Figure 4).

### 6.3. The IL-3 Family and Its Role in Megakaryopoiesis and Platelet Function

The IL-3 family includes IL-3 and IL-5, both of which employ a β-signaling receptor subunit to form a heterodimeric complex, thus classifying them as members of the β common chain cytokine family alongside GM-CSF [88]. This family of cytokines is initially identified for its potent pro-hematopoietic activity, stimulating bone marrow cell proliferation and multicolony formation and differentiation of hematopoietic cells. Subsequent studies have unveiled that this cytokine family plays significant roles not only in pro-hematopoiesis but also in a myriad of physiological and pathological regulatory processes [89]. As a result, its multifaceted nature is gaining increasing attention in diverse research domains. IL-5 is a cytokine that specifically targets eosinophils. It primarily orchestrates the differentiation, maturation, and migration of eosinophils within the myeloid lineage and is involved in related physiological and pathological processes [90]. However, it does not play a definitive role in the differentiation of other lineages, including the MK lineage.

IL-3 exhibits a broad spectrum of pro-hematopoietic activities. It stimulates the proliferation and differentiation of early HSCs, as well as the growth of multi-lineage hematopoietic progenitor cells, both in vivo and in vitro [91,92,93,94]. Notably, unlike the other two cytokines, IL-3 possesses the capability to promote MK differentiation and platelet production [64,95]. Upon forming receptor complexes, IL-3 could activate several pathways beyond the primary JAK/STAT pathway. It can stimulate pathways such as RAS/RAF/MAPK and PI3K/AKT directly or indirectly. These activations play a range of physiological regulatory roles and serve as important pathways for promoting MK differentiation [89,96]. However, the ability of IL-3 to promote MK differentiation is not specific, and it primarily regulates the growth and function of mast cells and basophils. The findings indicate that IL-3, unlike other pro-hematopoietic factors such as IL-6, loses its capacity to stimulate MK differentiation and platelet production in TPO receptor-deficient mice. Furthermore, no exacerbation of deficiencies in MKs and platelets is observed in mice deficient in both MPL and IL-3Rα [51]. This implies that the capacity of IL-3 to encourage megakaryopoiesis might rely on the TPO/c-Mpl pathway. Furthermore, it has been reported that IL-3, which is significantly elevated in septic bodies and enhances bone marrow myelopoiesis, is also responsible for driving the increase in splenic megakaryopoiesis in septic mice and has a profound effect on splenic immune-skewed platelet production [97,98].

Previous clinical trials have demonstrated that IL-3 can elevate peripheral platelet and neutrophil counts and mitigate chemotherapy-induced myelosuppression in cancer patients suffering from bone marrow failure and undergoing chemotherapy. However, IL-3 could not reduce platelet transfusions in certain cancer patients and has not shown improvement in adherence to chemotherapy regimens [99]. Additionally, side effects such as fever, headache, and rash have been reported, which have restricted its usage in patients with clinical myelosuppression [100,101].

### 6.4. The IL-6 Family and Its Role in Megakaryopoiesis

**Figure 5:**
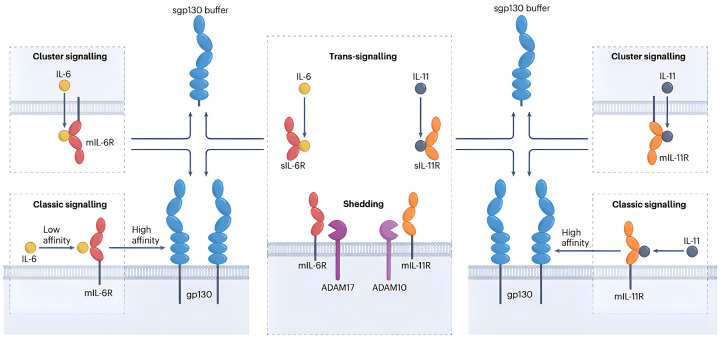
Signaling modalities of IL-6 and IL-11. Reproduced with permission from [103], copyright © 2023, Springer Nature Limited.

IL-6, a pleiotropic cytokine originally described for its role in B-cell differentiation and antibody production and now known for its wide range of biological activities, serves as an integral component of the IL-6 cytokine family, which also includes IL-11 [102]. IL-6 signaling involves the interaction with its specific receptor, IL-6R, and subsequently with gp130 to form a receptor complex, initiating signal transduction. Notably, IL-6 employs three distinct signaling modalities: classical signaling, involving binding to membrane-bound IL-6R-gp130; trans-signaling, wherein IL-6 first binds to soluble IL-6R before interacting with gp130; and cluster signaling, which involves the formation of an IL-6 receptor complex on the membrane of one cell that subsequently acts on the gp130 of another cell’s membrane [103] (Figure 5). These diverse signaling mechanisms contribute to the pleiotropic nature of IL-6. The IL-6 signaling pathway is implicated in the onset and progression of various diseases, including cancer, autoimmune disorders, and inflammatory diseases. Current clinical approaches to treating these diseases often involve inhibiting the IL-6 signaling pathway. However, research suggests that IL-6 trans-signaling is the primary pathway promoting these diseases [104]. Therefore, precise inhibition of this pathway is an active area of research.

IL-6 has been extensively studied for its role in promoting megakaryopoiesis both in vitro and in vivo. In vitro studies have shown that IL-6, in combination with other cytokines, significantly increases the number of hematopoietic progenitor cells, and IL-6 promotes the MKs maturation when used alone. In addition, immature human MKs have been observed to secrete IL-6, suggesting that IL-6 may contribute to MK maturation via autocrine secretion [47,105,106]. In vivo studies have shown that the thrombopoietic effect of IL-6 has been observed in various species, including mice, dogs, and primates. And those studies suggest that IL-6 primarily acts on differentiated MKs, promoting increases in their size, ploidy, and activity. This action promotes the maturation of MKs, leading to an increase in platelet counts. Interestingly, IL-6 appears to have no effect on MKP and MK counts [107,108,109]. Previous clinical studies have also demonstrated its role in promoting the maturation of MKs and the recovery of platelet levels in cancer patients following chemotherapy [110,111]. In terms of mechanisms, the formation of receptor complexes by IL-6 can activate multiple pathways to regulate the physiological and pathological processes of the organism. The JAK/STAT signaling pathway is the most significant among the pathways. IL-6 signaling can mediate the activation and phosphorylation of tyrosine residues in the tail of the JAK family of receptors, promoting the phosphorylation of STAT and the formation of homodimers that can be translocated to the nucleus. This process triggers multi-gene transcription, promoting MK proliferation and differentiation, among other effects [112,113]. It is noteworthy that IL-6 plays a pivotal role in the progression of several cancers, including numerous forms of leukemia, via this pathway. Further investigation might be warranted to explore methods of harnessing this pathway for promoting the proliferation and differentiation of MKs while avoiding its deleterious effects.

IL-11 belongs to the IL-6 cytokine family and displays pro-hematopoietic effects closely resembling those of IL-6. It plays a pivotal role in promoting the proliferation and differentiation of cells within the MK lineage, as well as the production of platelets. IL-11 could intervene in various stages of MK differentiation, including the proliferation of MKP and the maturation of MK [114]. The proliferative influence of IL-11 on MKPs is attributed to the synergistic interaction with other pro-hematopoietic factors in vitro. Administering IL-11 in isolation does not induce the expansion of MK colonies in human bone marrow mononuclear cells following T-cell depletion in vitro. The augmentation of both the quantity and size of MK colonies is solely facilitated by the conjoint effect of other early pro-hematopoietic factors, such as IL-3. However, the administration of IL-11 alone could enhance the ploidy of immature MKs, implying that IL-11 has a maturation-stimulating effect on MKs [115]. The administration of recombinant human interleukin-11 to mice exhibits a proliferative impact on diverse hematopoietic progenitor cells within their bone marrow, and it abbreviates the cell cycle of these progenitor cells. This effect is particularly significant in the MK lineage cells. Moreover, IL-11 expedites the reconstitution of bone marrow and hastens the restoration of peripheral neutrophil and platelet counts in mice experiencing myelosuppression following chemotherapy [116]. IL-11 and IL-6 exhibit largely analogous signaling mechanisms, each binding to either the membrane-bound or soluble IL-11R prior to associating with gp130 to form a receptor complex that subsequently initiates a signaling cascade. It is worth noting that the classical and trans-signaling pathways of IL-11 have been recognized in vivo, while the cluster signaling modality has been identified exclusively in vitro [117]. Similar to IL-6, IL-11 forms receptor complexes that predominantly trigger the JAK-STAT pathway, facilitating the translocation of STAT dimer to the nucleus and subsequently inducing the transcription of specific genes [118]. IL-11 exhibits significant thrombopoietic capacity, and unlike IL-6, which promotes the proliferation of a variety of cancer cells, the U.S. FDA has approved the clinical use of rhIL-11. rhIL-11 is now commonly used in clinical cancer patients with chemotherapy-induced thrombocytopenia and has been shown to be effective in reducing platelet transfusions in clinical patients [119].

### 6.5. The IL-17 Family and Its Role in Megakaryopoiesis and Platelet Function

The IL-17 family consists of six members, designated IL-17A to IL-17F. IL-17A, a ubiquitous pro-inflammatory factor, plays a pivotal role in regulating diverse physiological and pathological processes in the body. It exerts regulatory control over numerous cell types, including platelets, and has been implicated in the progression of certain cardiovascular diseases, particularly atherosclerosis and thrombosis [120,121]. IL-17A’s role in these pathologies is primarily attributed to its pro-platelet activity and function. Fundamental research has revealed the presence of IL-17A receptor mRNA within platelets. Moreover, IL-17A receptors are markedly amplified on the surface of activated platelets, suggesting that IL-17A may exert an influence on platelet functionality [70]. In vitro, IL-17A, in combination with ADP, significantly enhances platelet aggregation. However, IL-17A alone does not affect platelet aggregation. Therefore, IL-17A has been proposed as a potential synergistic factor that enhances ADP-induced platelet aggregation [122]. Additional mechanistic investigations have uncovered that IL-17A can stimulate platelet activation and aggregation by activating the platelet ERK2 signaling pathway and enhancing the phosphorylation level of platelet ERK2, and it has been found that ERK2-specific inhibitors can suppress this alteration induced by IL-17A [69,123]. Another study discovered that the expression of IL-17A in mice does not influence peripheral platelet counts. However, it does instigate an increase in bone marrow-derived CFU-MK and an expansion of mouse MK counts. Interestingly, this effect is not observed in c-MPL^−^/^−^ mice, implying that the pro-megakaryopoiesis role of IL-17A is contingent upon the c-Mpl [68]. IL-17A has been identified as a key factor mediating thrombosis in some diseases, but whether it has the ability to promote megakaryopoiesis and platelet activation in normal or thrombocytopenic individuals remains to be investigated.

### 6.6. Other Interleukins in Megakaryopoiesis and Platelet Function

IL-8, also known as CXCL8, is a chemokine of the CXC subfamily secreted by diverse cell types. Upon binding to its cognate receptors CXCR1 and CXCR2, IL-8 activates downstream pathways, including MAPK/ERK and PI3K/AKT pathways, triggering chemotaxis and activation of neutrophils, and is implicated in the pathogenesis of many diseases [124,125]. Elevated serum levels of IL-8 have been observed in patients with myeloid metaplasia with myelofibrosis and hematopoietic cells that contribute to the production of IL-8. Further studies have revealed that suppression of IL-8 expression in patients augments the proliferation of CD34+ stem cell-derived cells and stimulates their differentiation into MKs. Additionally, anti-IL-8 treatment enhances the expression of markers associated with MK differentiation and maturation, increasing the number of multinucleated MKs. These findings suggest that endogenous IL-8 exerts a negative regulatory effect on the proliferation and maturation of MKs [126]. Another study shows that inhibiting the secretion of IL-8 and its receptor, CXCR2 significantly enhances lineage commitment and proliferation of MKPs derived from cord blood HSPCs. This mechanism appears to be related to the upregulation of several key transcription factors involved in MK development following the inhibition of IL-8 [71]. With respect to platelet functionality, in vitro studies have shown that anti-IL-8 antibodies can stimulate platelet activation and enhance platelet coagulation activity in patients with heparin-induced thrombocytopenia. However, the exact mechanism underlying this phenomenon remains to be elucidated [72].

IL-13 and IL-4 are closely related cytokines that play a critical role in modulating diseases associated with allergic inflammation. These cytokines can bind to their individual receptors and subsequently to each other, forming the IL-4/IL-13 receptor complex. This complex executes specific functions via multiple signaling pathways, including the STAT6 pathway [127]. IL-13 has also been found to promote megakaryopoiesis. Research indicates that IL-13 can promote the growth of human MKPs alone. Furthermore, it enhances the formation of MK colonies induced by IL-3 [128]. MKs from mice with myelofibrosis models exhibit high expression levels of IL-13Rα1 and IL-4Rα, along with increased expression of IL-13 in bone marrow. Subsequent investigations have revealed that IL-13 can instigate the expansion and growth of mutant MKs in mice with myelofibrosis models. This process appears to promote the progression of myelofibrosis via the activation of the STAT6 pathway [129].

In summary, the mechanisms of interleukin effects on MK lineage cells cover many aspects of MK differentiation and platelet production and function and involve a variety of pathways such as JAK/STAT, MAPK/ERK, PI3K/AKT, and so on. Although the physiological conditions at the time of the study are different, most interleukins, such as IL-1, IL-3, IL-6, IL-9, IL-11, IL-13, IL-17A, etc., have a promoting effect on the differentiation and function of MK lineage cells, with IL-2 and IL-33 acting mainly indirectly. And some interleukins, such as IL-4, IL-8, and IL-37, have negative regulatory effects on MK lineage cells. In particular, IL-21 has a bidirectional regulatory effect on platelet production. In addition, earlier interleukins have been better studied, such as rhIL-3 and rhIL-6, have been studied in vivo in a variety of mammalian species, including dogs, non-human primates, and mice, and have shown consistent effects in promoting MK differentiation and thrombopoiesis, lending more credence to the effects of these interleukins [66,130,131]. These mechanistic studies provide important clues for understanding the biological mechanisms of platelets and for developing new therapeutic strategies, especially for platelet-related diseases, but further studies are needed to validate their value for clinical applications.

## 7. Conclusions and Prospects

The discovery and characterization of interleukins in the 1980s and 1990s marked a significant breakthrough in our understanding of immune regulation and hematopoiesis. With the potential to modulate these processes, interleukins held promise as therapeutic agents for various diseases. However, despite extensive research, the clinical application of interleukins as pharmacological agents has been met with challenges. While interleukins play a crucial role in megakaryopoiesis, their pleiotropic nature, non-specific targeting, and short half-life have hindered their systematic development as therapeutic agents. Clinical trials have demonstrated the difficulty in harnessing the beneficial effects of interleukins while minimizing their adverse effects. Among the interleukins discussed in this review, only rhIL-11 has been approved for clinical use in thrombopoiesis. Current clinical research has shifted toward the development of biological inhibitors of interleukins, targeting specific interleukins and their pathways involved in disease development. These inhibitors have shown promising results in terms of safety and efficacy, but their limitations highlight the untapped therapeutic potential of interleukins [132].

**Figure 6:**
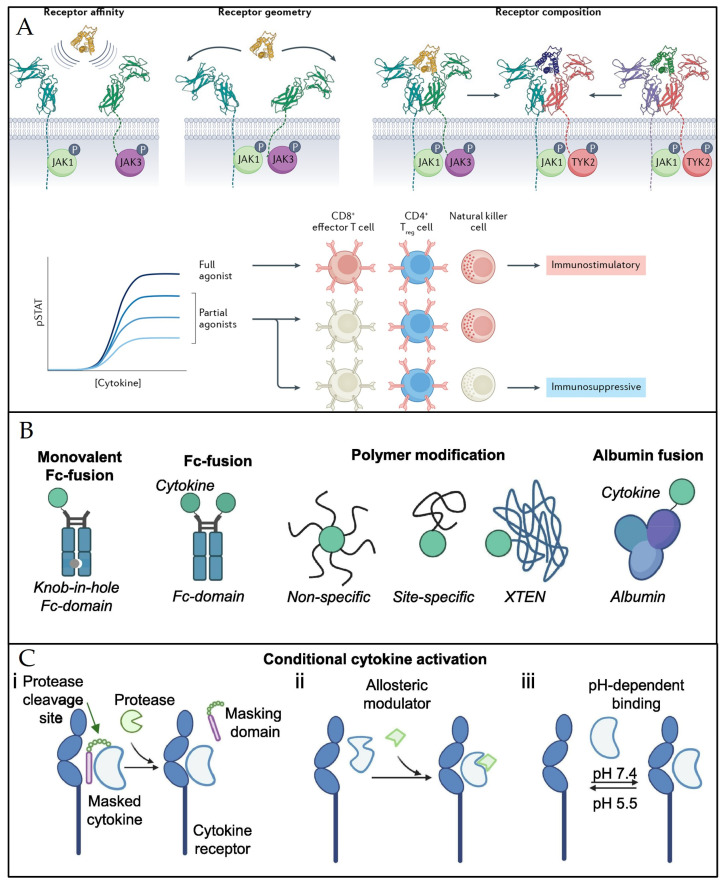
Schematic diagram of cytokine engineering methods to improve the druggability of interleukins. Bioengineering techniques modify cytokine receptor affinity, geometry, and composition to regulate cytokine effects (**A**). Reproduced with permission from [133], copyright © 2022, Springer Nature Limited. Multiple approaches to modifying cytokines to achieve conditional activation of cytokines (**B**); multiple methods to augment the molecular mass of cytokines for prolonging their lifespan (**C**). Reproduced with permission from [134], © 2022 The Author(s). Published by Elsevier B.V.

The challenges associated with interleukin therapy stem from their inherent properties and the complexities of the immune system. A significant portion of research on interleukins and MK lineage cells has focused on understanding the consequences of disease-induced interleukin augmentation, primarily within the context of the immune system. Pathological conditions often involve elevated levels of endogenous interleukins and immune system activation. Similarly, the administration of exogenous interleukins, which also stimulate the immune system, can induce undesired immune responses such as systemic inflammation and allergic reactions. These limitations pose significant hurdles to the clinical use of interleukins. The fundamental characteristics of interleukins that impede their utilization as cytokine therapeutics include the following: (i) pleiotropy: Interleukins can exert a variety of effects, and their role can vary depending on the specific conditions. This multifaceted nature makes it challenging to predict and control the therapeutic effects of interleukins; (ii) non-targeting: Interleukins can affect diverse cell types without specific targeting. While the local secretion of endogenous interleukins can impact adjacent tissues and cells, targeting specific sites with exogenous interleukins remains challenging, often resulting in non-targeted or systemic cytotoxicity; and (iii) short half-life: Due to their small molecular weight and short half-life, interleukins require continuous administration to maintain their effectiveness. However, this continuous administration can lead to the accumulation of side effects or toxicity. Fortunately, these challenges are being addressed via ongoing advancements in cytokine engineering. A multitude of technologies are being developed to overcome the limitations of interleukins and harness their therapeutic potential. To address the issue of pleiotropy, cytokine engineering techniques enable the manipulation of essential pharmacological parameters of interleukin-receptor binding. These adjustments include changes in interleukin-receptor affinity, receptor geometry, and receptor subunit composition. These modifications allow for the regulation of the level of interleukin signaling activation, enabling interleukins to produce varying effects that align with the desired clinical therapeutic objective (Figure 6A). Regarding targeting, conditional activation between cytokines and receptors can be achieved via cytokine modification. This means that cytokines can only bind to receptors under specific physiological conditions to perform their functions. For instance, modified cytokines may bind to receptors in the presence of specific proteases, allosteric modulators, or pH environments. This targeted approach allows cytokines to reach the disease site while minimizing systemic effects (Figure 6B). Finally, cytokines can be combined with other small molecules or polymers, such as the FC structural domain of IgG, polyethylene glycol (PEG), and albumin, using various methods. This process increases the molecular weight of the target cytokine, extending its half-life while also enhancing its stability and reducing its metabolic rate [133,134] (Figure 6C).

Beyond the aforementioned techniques, various bioengineering approaches are being explored to improve the druggability of cytokines like interleukins. Currently, research on the bioengineering of interleukins is well advanced and maturing in the field of cancer therapy; some of the results are being translated into the clinic, while research is also being developed in other areas of disease treatment [135]. The clinical implementation of these next-generation cytokine therapies holds considerable promise for the treatment of various diseases.

## Figures and Tables

**Table 1:** Effects of interleukins on megakaryopoiesis and platelet function.

Interleukins	Cell Sources	Effect on Megakaryopoiesis	Effect on Platelet Function	Refs.
**IL-1 Family**				
IL-1α	Macrophages Monocytes Microglia MKs, etc.	Inducing MK rupture for rapid platelet production; inducing MK differentiation and maturation; inhibiting PPF formation.	/	[49]
IL-1β	Consistent with IL-1α	Promoting MK differentiation and maturation; inducing thrombopoiesis.	Enhancement of MK and platelet function.	[53,54]
IL-33	Epithelial cells Necrotic cells Fibroblasts, etc.	/	Indirect promotion of platelet activation via 5-HT.	[55]
IL-37	Monocytes Tonsil plasma cells Breast carcinoma cells, etc.	/	Inhibition of platelet activation and thrombosis in vivo.	[56]
**IL-2 Family**				
IL-2	CD4+ and CD8+ T cells DCs NK and NKT cells, etc.	Indirect increasing platelet counts in ITP patients; reducing platelet count in cancer patients.	Enhancement of platelet viability.	[57,58]
IL-4	T_H_2 cells, Basophils, Eosinophils, Mast cells, etc.	Inhibiting MK differentiation at all stages.	/	[59,60]
IL-9	T_H_2, T_H_9, T_H_17 cells Treg cells Mast cells, etc.	Promoting MK maturation and platelet production.	Promotion of platelet function and thrombosis	[61,62]
IL-21	T cells NKT cells, etc.	Promoting the proliferation of MKP and MK differentiation; increasing platelet clearance.	/	[63]
**IL-3 Family**				
IL-3	T cells Macrophages NK cells Mast cells, etc.	Promoting MKP proliferation and MK differentiation	/	[64]
**IL-6 Family**				
IL-6	Monocytes/macrophages Endothelial cells Granulocytes Osteoblasts, etc.	Promoting MK maturation and thrombopoiesis; enhancing the growth-promoting effect of TPO on MKs	Enhancement of canine platelet sensitivity to thrombin and platelet-activating factors	[65,66]
IL-11	Bone marrow cells Epithelial cells Endothelial cells Osteoblasts, etc.	Increasing bone marrow and splenic progenitor cell counts; promoting MK maturation to increase platelet counts	/	[67]
**IL-17 Family**				
IL-17A	TH17 cells CD8+ T cells NK cells, etc.	Amplifying bone marrow-derived CFU-MK; regulating and synergizing the TPO/c-Mpl pathway for platelet function and factor release	/	[68,69,70]
**Others**				
IL-8	Monocytes Macrophages Neutrophils Lymphocytes Endothelial cells, etc.	Negative regulating MK lineage differentiation of HSPCs and proliferation of MKP	Negative regulation of platelet activation in patients with heparin-induced thrombocytopenia	[71,72]
IL-13	NKT cells Mast cells Basophils Eosinophils, etc.	Stimulating MK colony formation; promoting MK differentiation in HEL cells and Dami cells	/	[73]

## Data Availability

Data sharing is not applicable.
